# Resistant Hypertension in Patients With Type-2 Diabetes Mellitus: A Single-Center, Cross-Sectional Study

**DOI:** 10.7759/cureus.30228

**Published:** 2022-10-12

**Authors:** Aqeel S Binaqeel, Hossein A Filimban, Abdullah A Fallatah, Salman W Bafageeh, Sara H Al Khansa, Bader K Al Aslab, Rayan S Alzahrani, Leyan R Bakedo, Ahmed Abuosa, Ibrahim Jelaidan

**Affiliations:** 1 College of Medicine, King Saud Bin Abdulaziz University for Health Sciences, Jeddah, SAU; 2 Pharmaceutical Care Services, King Abdulaziz Cardiac Center, National Guard Health Affairs, Jeddah, SAU; 3 Medicine and Surgery, King Abdulaziz University, Jeddah, SAU; 4 Cardiology, National Training Institute, Cairo, EGY; 5 Cardiology, King Abdulaziz Cardiac Center, National Guard Health Affairs, Jeddah, SAU

**Keywords:** hypertension, prevalence, ischemic heart disease, antihypertensives, type-2 diabetes mellitus, resistant hypertension

## Abstract

Background

The prevalence of resistant hypertension in Saudi patients with type-2 diabetes mellitus (T2DM) has not been previously estimated. Therefore, our objective was to assess the prevalence and characteristics of resistant hypertensive patients with T2DM at King Abdulaziz Medical City, Jeddah, Saudi Arabia.

Methods

This cross-sectional study included patients with hypertension and T2DM who presented to our center in 2018. We examined 1960 patients with T2DM during the study period; 809 were hypertensives. We compared T2DM patients with controlled hypertension versus resistant hypertension.

Results

The prevalence of resistant hypertension in patients with T2DM was 137/809 (16.93%). The mean age was 66.38±10.80 years, and females presented 56% of the study population (n= 451). Obstructive sleep apnea (OSA; OR: 2.60 [1.15- 5.87]; P=0.02) and ischemic heart disease (IHD; OR: 3.01 [2.04- 4.45]; P˂0.001) were significantly associated with resistant hypertension. The most common medications used with resistant hypertension were calcium channel blockers (CCBs; 89.05%), β-blockers (76.64%), and angiotensin-2 receptor blockers (ARBs; 62.77%).

Conclusions

Resistant hypertension in patients with T2DM is common in Saudi Arabia. Resistant hypertension could be associated with OSA and IHD. Further studies are required to evaluate the temporal relationship between resistant hypertension and risk factors.

## Introduction

Hypertension is a significant cause of morbidity and mortality globally, impacting an estimated 1.4 billion people [1,2]. Chronic hypertension increases the risk of heart attack, stroke, heart failure, renal disease, and mortality if left untreated, presenting major health concerns to patients and healthcare providers [2]. The European Society of Cardiology defines resistant hypertension as the failure of three or more antihypertensive medications to lower systolic and diastolic blood pressure values to <140 mmHg and/or <90 mmHg, respectively [3]. The inadequate control of blood pressure is confirmed by ambulatory blood pressure monitoring or home blood pressure monitoring in patients adherent to therapy [3]. Furthermore, the American College of Cardiology stated that controlled hypertension using at least four medications is also considered resistant hypertension [4]. The recommended treatment strategy for patients with resistant hypertension includes appropriate lifestyle measures and treatment with optimal or best-tolerated doses of three or more drugs. These medications should consist of diuretics, angiotensin-converting enzyme inhibitors (ACEi), angiotensin-2 receptor blockers (ARBs), and calcium channel blockers (CCBs) [3].

The prevalence of resistant hypertension with type 2 diabetes mellitus (T2DM) varies in different countries. A study published in 2015 estimated that the prevalence of resistant hypertension was 10% in Romania [5]. Moreover, according to the National Health and Nutrition Examination Survey (NHANES), the prevalence of resistant hypertension was 8.9% in the United States between 2003 and 2008, and has increased to 13% in 2017 [6,7]. The estimated prevalence of T2DM in Saudi Arabia is 23.7% [8], and hypertension is 33% [9]. However, there is no data on resistant hypertension prevalence in patients with type 2 diabetes and their characteristics in Saudi Arabia. Thus, we aimed to assess the prevalence and characteristics of resistant hypertensive patients with T2DM at King Abdulaziz Medical City (KAMC), Jeddah, Saudi Arabia.

## Materials and methods

Study design and patients

This cross-sectional study included patients with hypertension and T2DM attending King Faisal Cardiac Center in King Abdulaziz Medical City, Jeddah, Saudi Arabia, in 2018. We included patients 18 years old and older who visited the outpatient clinics of the internal medicine and cardiology departments in 2018. Patients with heart failure and reduced ejection fraction (<40%) were excluded as they would be on three types of antihypertensive medications. We examined 1960 patients with T2DM during the study period; 809 were hypertensives. The study was approved by the Institutional Review Board (IRB) of King Abdullah International Medical Center with the number (NRJ22J/127/05), and the need for the patient's consent was waived.

Data and definitions

Resistant hypertension was diagnosed according to the European guidelines if three or more medications failed to control the blood pressure and the patients adhered to their antihypertensive medications [3]. We reviewed electronic charts to collect patients' data. Baseline data included age, gender, body mass index (BMI), and systolic and diastolic blood pressure. Additionally, we collected data related to the concomitant comorbidities based on the history provided in the patient's electronic medical records, including ischemic heart disease (IHD) defined by patients who underwent percutaneous coronary intervention or coronary artery bypass graft, OSA, chronic kidney disease (CKD), pheochromocytoma, Cushing syndrome, hypothyroidism, and hyperthyroidism. The number of antihypertensive medications and their categories was reported.

Statistical analysis

We compared patients' related data between those with controlled versus resistant hypertension with T2DM. Continuous variables were assessed for normality using the Shapiro-Wilk test and histograms. In addition, the student t-test was used to compare normally distributed data, and the Mann-Whitney test for non-normally distributed continuous data. Numerical data were presented as mean and SD if normally distributed and median (25th-75th percentiles) if not normally distributed. Categorical data were presented as absolute numbers and percentages and compared with the Chi-square or Fisher's exact test when appropriate.

Multivariable logistic regression was used to identify factors associated with resistant hypertension. Univariable regression was performed, and variables with a P-value of 0.15 or less were introduced into a stepwise multivariable regression analysis with backward elimination. Factors associated with resistant hypertension were divided into two models: model 1 included patients' demographics and comorbidities, and model 2 had antihypertensive medications. Model calibration was tested using the Hosmer-Lemeshow test and model discrimination with the C-statistics and area under the curve. Multicollinearity was tested using variance inflation factor (VIF), and all factors included in the final multivariable regression model had a VIF of 1.5 or less.

All analyses were performed using Stata 16.1 (StataCorp, TX, USA), and a p-value of less than 0.05 was considered statistically significant.

## Results

Prevalence of resistant hypertension among T2DM

Among patients with T2DM and concomitant hypertension, the prevalence of resistant hypertension was estimated to be 137/809 (16.93%).

Baseline characteristics

The mean age was 66.38±10.80 years, with no difference between patients with resistant and controlled hypertension. Females presented 56% of the study population (n= 451). There were no differences in gender, BMI, and HbA1c between groups. Patients with T2DM and resistant hypertension had a higher prevalence of OSA, CKD, and IHD than those with T2DM and non-resistant hypertension. Neither group had significant differences in smoking, HbA1c, and endocrine disease. In both groups, no patient was affected with pheochromocytoma, Cushing syndrome, or renal artery stenosis (Table 1).

**Table 1:** Comparison of the baseline characteristics and comorbidities between type 2 diabetes patients with controlled versus resistant hypertension. CKD: Chronic kidney disease; IHD: Ischemic heart disease; OSA: Obstructive sleep apnea.

	Total (n=809)	Controlled HTN (n=672)	Resistant HTN (n=137)	P-value
Age (years)	66.38± 10.80	66.22± 10.84	67.20± 10.61	0.33
Female	451 (55.75%)	375 (55.80%)	76 (55.47%)	0.94
Systolic pressure (mmHg)	138 (127.3-147)	135 (125.32-144.80)	148.30 (143.60-153.60)	˂0.001
Diastolic pressure (mmHg)	67 (60-75)	67 (60-74.32)	69 (60.6-77.6)	0.13
BMI (Kg/m2)	31.63 (27.81-36.19)	31.54 (27.79-36.04)	32.46 (27.92-36.79)	0.44
HbA1c (%)	7.3 (6.4-8.6)	7.3 (6.4-8.6)	7.1 (6.4-8.8)	0.87
Smoking	52 (6.43%)	39 (5.80%)	13 (9.49%)	0.11
OSA	29 (3.58%)	19 (2.83%)	10 (7.30%)	0.01
CKD	115 (14.22%)	86 (12.80%)	29 (21.17%)	0.01
IHD	193 (23.86%)	134 (19.94%)	59 (43.07%)	˂0.001
Hyperaldosteronism	1 (0.12%)	0	1 (0.73%)	0.17
Hypothyroidism	128 (15.82%)	106 (15.77%)	22 (16.06%)	0.93
Hyperthyroidism	8 (0.99%)	7 (1.04%)	1 (0.73%)	˃0.99

Medications

The most common medications used were ARBs (47.71%), CCBs (46.97%), and β-blockers (36.96%). In patients with resistant hypertension, the most common medications were CCBs (89.05%), β-blockers (76.64%), and ARBs (62.77%). Diuretics (P˂0,001), mineralocorticoid receptor antagonists (MRA) (P= 0.01), CCBs (P˂0.001), ARBs (P ˂0.001), β-blockers (P˂0.001), and vasodilators (P˂0.001) were used more frequently in patients with resistant hypertension compared to controlled hypertension. However, there were no differences in ACEi, non-steroidal anti-inflammatory drugs (NSAIDs), and steroids use between groups (Table 2).

**Table 2:** Comparison of the medications between type 2 diabetes patients with controlled versus resistant hypertension. ACEi: Angiotensin-converting enzyme inhibitors; ARBs: Angiotensin receptor blocker; CCBs: Calcium channel blockers; MRA: Mineralocorticoid receptor antagonist; NSAIDs: Non-steroidal anti-inflammatory drugs.

	Total (n=809)	Controlled HTN (n=672)	Resistant HTN (n=137)	P-value
Diuretics	156 (19.28%)	93 (13.84%)	63 (45.99%)	˂0.001
MRA	22 (2.72%)	14 (2.08%)	8 (5.84%)	0.01
CCBs	380 (46.97%)	258 (38.39%)	122 (89.05%)	˂0.001
ACEi	243 (30.93%)	202 (30.06%)	41 (29.93%)	0.98
ARBs	386 (47.71%)	300 (44.64%)	86 (62.77%)	˂0.001
β-blockers	299 (36.96%)	194 (28.87%)	105 (76.64%)	˂0.001
α1-blockers	1 (0.12%)	1 (0.15%)	0	˃0.99
Vasodilators	49 (6.06%)	22 (3.27%)	27 (19.71%)	˂0.001
NSAIDs	94 (11.62%)	77 (11.46%)	17 (12.41%)	0.75
Steroids	32 (3.96%)	26 (3.87%)	6 (4.38%)	0.78
Number of HTN medications				
Zero	44 (5.44%)	44 (6.55%)	0	˂0.001
One	241 (29.79%)	241 (35.86%)	0	
Two	333 (41.16%)	333 (49.55%)	0	
Three	152 (18.79%)	52 (7.74%)	100 (72.99%)	
Four	37 (4.57%)	2 (0.30%)	35 (25.55%)	
Five	2 (0.25%)	0	2 (1.46%)	

Factors associated with resistant hypertension

Among the comorbidities associated with T2DM and resistant hypertension, OSA (OR: 2.60 [1.15- 5.87]; P= 0.02) and IHD (OR: 3.01 [2.04- 4.45]; P˂0.001) were significantly associated with resistant hypertension (Table 3).

**Table 3:** Multivariable logistic regression models for factors associated with resistant hypertension. *Model 1: Hosmer-Lemeshow, P=0.07, AUC=0.63. **Model 2: Hosmer-Lemeshow, P=0.07, AUC=0.95. ¥ Number of medications excluded for collinearity CKD: Chronic kidney disease; IHD: Ischemic heart disease; OSA: Obstructive sleep apnea; ACEi: Angiotensin-converting enzyme inhibitors; ARB: Angiotensin receptor blocker; CCBs: Calcium channel blockers; MRA: Mineralocorticoid receptor antagonist; NSAIDs: Non-steroidal anti-inflammatory drugs.

	Univariable		Multivariable	
	OR (95% CI)	P-value	OR (95% CI)	P-value
*Model 1				
Age	1.01 (0.99-1.03)	0.33		
Gender	1.01 (0.70-1.47)	0.94		
BMI	1.01 (0.98-1.03)	0.69		
HbA1c (%)	1.001 (0.90-1.11)	0.99		
Smoking	1.70 (0.88-3.28)	0.11	-	-
OSA	2.70 (1.22-5.96)	0.01	2.60 (1.15-5.87)	0.02
CKD	1.83 (1.15-2.92)	0.01	-	-
IHD	3.04 (2.06-4.47)	˂0.001	3.01 (2.04-4.45)	˂0.001
Hypothyroidism	1.02 (0.62-1.69)	0.93		
Hyperthyroidism	0.70 (0.09- 5.72)	0.74		
**Model 2				
Diuretics	5.30 (3.55-7.92)	˂0.001	83.95 (29.47- 239.08)	˂0.001
MRA	2.91 (1.20-7.09)	0.02	15.62 (2.82- 86.52)	0.002
CCBs	13.05 (7.47-22.81)	˂0.001	85.42 (29.93- 243.80)	˂0.001
ACEi	0.99 (0.67-1.48)	0.98		
ARBs	2.09 (1.43-3.05)	˂0.001	-	-
β-blockers	8.08 (5.26-12.42)	˂0.001	81.75 (29.47-226.78)	˂0.001
Vasodilators	7.25 (3.99-13.19)	˂0.001	33.72 (10.36-109.80)	˂0.001
NSAIDs	1.09 (0.62-1,92)	0.75		
Steroids	1.14 (0.46-2.82)	0.78		
¥ Number of medications	297.11 (71.87-1228.29)	˂0.001	-	-

## Discussion

Hypertension is a significant cause of morbidity and mortality worldwide [1]. However, the prevalence of resistant hypertension in patients with T2DM in Saudi Arabia was not estimated before, despite the high prevalence of diabetes and hypertension [8,9]. This retrospective cross-sectional study estimated the prevalence of resistant hypertension in T2DM patients presenting to a single center in 2018. The prevalence was 16.93% (137/809), and most patients were females. ARBs, CCBs, and β- blockers were the most common medications used in all patients, while CCBs, β-blockers, and ARBs were the most common medications in patients with resistant hypertension. In addition, IHD, CKD, and OSA were significantly higher in patients with resistant hypertension.

Our study's prevalence of resistant hypertension in patients with T2DM was consistent with other studies. Solini A et al. reported a prevalence of 17% in Italy [10], and Mohammad A et al. reported a prevalence of 16% in Jordan [11]. These articles reported a significant association between resistant hypertension and T2DM. In a meta-analysis by Achelrod D et al., the prevalence of resistant hypertension ranged from 13% to 16% [12]. Noubiap JJ et al. estimated the prevalence of true resistant hypertension in a meta-analysis of 3.2 million patients to be 10%, and the prevalence differed significantly according to the associated disease [13]. The variability of the prevalence of resistant hypertension in different studies could be attributed to several factors, including the study design, definitions used, the settings used to measure blood pressure, and the associated comorbidities.

Our study evaluated factors associated with resistant hypertension in patients with T2DM. IHD and OSA were significantly associated with resistant hypertension in our cohort. A study reported that 14.1% of resistant hypertensive patients had associated IHD [14]. This percentage is remarkably less than our population, in which 43.07% of resistant hypertension patients had IHD. On the other hand, Mohammad A et al. reported that 54.6% (77/141) of resistant hypertension patients had IHD [11]. Our data resource could explain our results since most data were from a cardiac center. In regards to OSA, a multi-center study on OSA patients with resistant hypertension by Sapiña-Beltrán E et al. estimated that 237/284 (83.6%) had resistant hypertension and OSA [15]. In addition, approximately 47.2% (112/237) of those patients had concomitant T2DM [15]. In our data, only 7.30% (10/137) of patients had OSA with resistant hypertension and T2DM. The inconsistency of our data to the Sapiña-Beltrán E et al. study could be due to the limited availability of sleep study centers to diagnose OSA within our population. Furthermore, in contrast to the literature, CKD was not associated with resistant hypertension in our series by the multivariable regression analysis [10,13,14]. In our study, the CKD diagnosis was not based on estimated glomerular filtration rate measurement but the history available in the patient's electronic medical files, making our data susceptible to recall bias. 

ACEi, ARBs, and diuretics were the most frequently used medications to manage resistant hypertension, followed by CCBs and β-blocker [16]. Despite not being a first-line antihypertensive drug, β-blockers were our patient's second most-used drug. Similar results were found in a study by Ciobanu DM et al. [5]. This increased use of β-blockers is most likely linked to our study's high prevalence of IHD.

Further exploration of the causation and association between resistant hypertension and other risk factors is needed to implement new strategies and plans for intervention. In addition, better diagnostic tools to differentiate between true and pseudo-resistant hypertension should be prioritized to decrease unnecessary intervention and improve the quality of care. Finally, the discrepancy in resistant hypertension prevalence across different regions is small. Thus, creating an intervention that could be implemented in different settings regardless of socioeconomic status is possible [14].

Study limitations

The study has several limitations, including the study design. The retrospective design has its inherent limitations of selection, recall, and reporting biases. Additionally, the study is cross-sectional; therefore, the temporal relationship between risk factors and resistant hypertension could not be established. Moreover, the study is a single-center experience, mainly from a cardiac center. Including other centers and different healthcare settings could significantly change the results. Furthermore, more robust criteria for the comorbidities could yield better insights. Finally, we were not able to stage CKD or OSA due to the recent implementation of the electronic data system in our center, which led to data deficiency as not all data were transferred to the new system.

## Conclusions

Resistant hypertension in patients with T2DM is common in Saudi Arabia. Resistant hypertension could be associated with OSA and IHD. Further studies are required to evaluate the temporal relationship between resistant hypertension and risk factors.
